# Metabolic Versatility and Antibacterial Metabolite Biosynthesis Are Distinguishing Genomic Features of the Fire Blight Antagonist *Pantoea vagans* C9-1

**DOI:** 10.1371/journal.pone.0022247

**Published:** 2011-07-15

**Authors:** Theo H. M. Smits, Fabio Rezzonico, Tim Kamber, Jochen Blom, Alexander Goesmann, Carol A. Ishimaru, Jürg E. Frey, Virginia O. Stockwell, Brion Duffy

**Affiliations:** 1 Swiss National Competence Center for Fire Blight, Division of Plant Protection, Agroscope Changins-Wädenswil ACW, Wädenswil, Switzerland; 2 CeBiTec, Bielefeld University, Bielefeld, Germany; 3 Department of Plant Pathology, University of Minnesota, St. Paul, Minnesota, United States of America; 4 Department of Botany and Plant Pathology, Oregon State University, Corvallis, Oregon, United States of America; J. Craig Venter Institute, United States of America

## Abstract

**Background:**

*Pantoea vagans* is a commercialized biological control agent used against the pome fruit bacterial disease fire blight, caused by *Erwinia amylovora*. Compared to other biocontrol agents, relatively little is currently known regarding *Pantoea* genetics. Better understanding of antagonist mechanisms of action and ecological fitness is critical to improving efficacy.

**Principal Findings:**

Genome analysis indicated two major factors contribute to biocontrol activity: competition for limiting substrates and antibacterial metabolite production. Pathways for utilization of a broad diversity of sugars and acquisition of iron were identified. Metabolism of sorbitol by *P. vagans* C9-1 may be a major metabolic feature in biocontrol of fire blight. Biosynthetic genes for the antibacterial peptide pantocin A were found on a chromosomal 28-kb genomic island, and for dapdiamide E on the plasmid pPag2. There was no evidence of potential virulence factors that could enable an animal or phytopathogenic lifestyle and no indication of any genetic-based biosafety risk in the antagonist.

**Conclusions:**

Identifying key determinants contributing to disease suppression allows the development of procedures to follow their expression *in planta* and the genome sequence contributes to rationale risk assessment regarding the use of the biocontrol strain in agricultural systems.

## Introduction

Fire blight is the most important global threat to pome fruit production areas around the world and its causative agent, the enterobacterium *Erwinia amylovora*, can affect a wide variety of rosaceous plants [1]. During spring, the pathogen colonizes the stigmata of flowers and invades tissues through the nectaries, causing rapid necrosis and progressive wilt in infected branches. Epidemics can develop rapidly and result in death of individual plants or entire orchards within a single season, leading to severe economic losses [2]. *E. amylovora* has quarantine status outside North America, and it is a contentious trade issue with fruit movement into fire-blight-free countries such as Australia, Japan, and all countries of the Southern Hemisphere aside from New Zealand [3], [4]. This invasive pathogen has spread across Europe and the Middle East, and is threatening to advance into the region of origin of apple germplasm resources in Central Asia [5], adding to the urgency to develop more effective control and containment strategies. With the recent publication of the genomes of *E. amylovora* CFBP 1430 [6] and its close relative *Erwinia pyrifoliae* DSM 12163^T^ [7], a solid knowledge base for the pathogen is present already.

Although fire blight is one of the most intensively studied bacterial plant diseases, the control of the disease is still not satisfactory [8]. The most effective control of fire blight was obtained with prophylactic applications of the antibiotic streptomycin on flowers. Unfortunately, resistant strains of *E. amylovora* have emerged in production areas where streptomycin is registered for use [9]. Biological control of the disease has been tested over the last decades as a valuable alternative [8]. Here, several modes of action have been described. First, competition for space and nutrients is one of the most common mechanisms [10]. Acidification of the habitat can also produce unfavorable conditions for pathogen multiplication [11]. Additionally, antibiotic production by the biological control agent can suppress growth of the pathogen [12], [13].

*P. vagans* strain C9-1 [14], [15], [16] is an important biocontrol agent against *E. amylovora* [8], [17] that is registered in the USA and Canada as BlightBan C9-1S (NuFarms America). Two *Pantoea agglomerans* strains (E325 and P10c) are also commercially marketed for fire blight control. An obstacle to wider approval of *Pantoea* for agricultural application as a biocontrol agent is the reporting of clinical isolates, typically with specious documentation and/or identification, within the same species as the biocontrol strains. As a result, European and other governmental regulators have categorized most *Pantoea* spp. as biosafety level 2 (BL-2) organisms (opportunistic pathogens). This qualification of *Pantoea* spp. as BL-2 organisms restricts beneficial uses much needed as alternatives to even more controversial products (e.g., antibiotics) or to fill gaps where no other protection options are available. Detailed analysis of the complete genome sequence of the *P. vagans* biocontrol strain C9-1 [18] offers an important foundation for demonstrating biosafety and moreover for elucidating traits that influence and can ultimately be harnessed to improve beneficial biocontrol performance.

## Results

### Metabolic versatility in carbon metabolism

Nutrient competition by depriving pathogens of necessary resources on flowers is an important mechanism of action for biocontrol strains [10], [19]. We evaluated the metabolic versatility of *P. vagans* C9-1 with Biolog GN2 and AN plates, and Biolog PM profiling (Supporting Information, Table S1). *P. vagans* C9-1 metabolized a wide range of carbohydrates, corresponding well with already published substrate ranges for this organism [14], [20]. Many PTS systems are found in the genome annotation [18], most of them encoding sugar phosphorylases and most of them in close association to sugar-converting enzymes (e.g., kinases, glucosidases, etc). Additionally, some families of MFS and ABC transporters are predicted to transport sugars. The complete pathways including uptake proteins for several of these sugars were identified, providing genetic bases for most substrates found to be catabolized with Biolog PM profiling (Supporting information, Table S1).

On pome fruit hosts that utilize sorbitol as a primary carbon transport and storage compound [21], metabolism of sorbitol may contribute to virulence of *E. amylovora* [22], [23]. The genome of *P. vagans* C9-1 encodes two gene clusters for sorbitol utilization on plasmid pPag2 [18], a feature that is absent in other *P. vagans* strains [14]. At the protein level, the pPag2-encoded genes are 71–95% identical to each other, and 53–88% identical to the proteins encoded by the sorbitol gene clusters of *E. amylovora* [6]. Growth of *P. vagans* C9-1 with sorbitol as sole carbon source was confirmed with the Biolog plates (Supporting Information, Table S1) and in liquid cultures [15], [20].

### Biosynthesis of the antibiotic pantocin A

**Figure 1:**
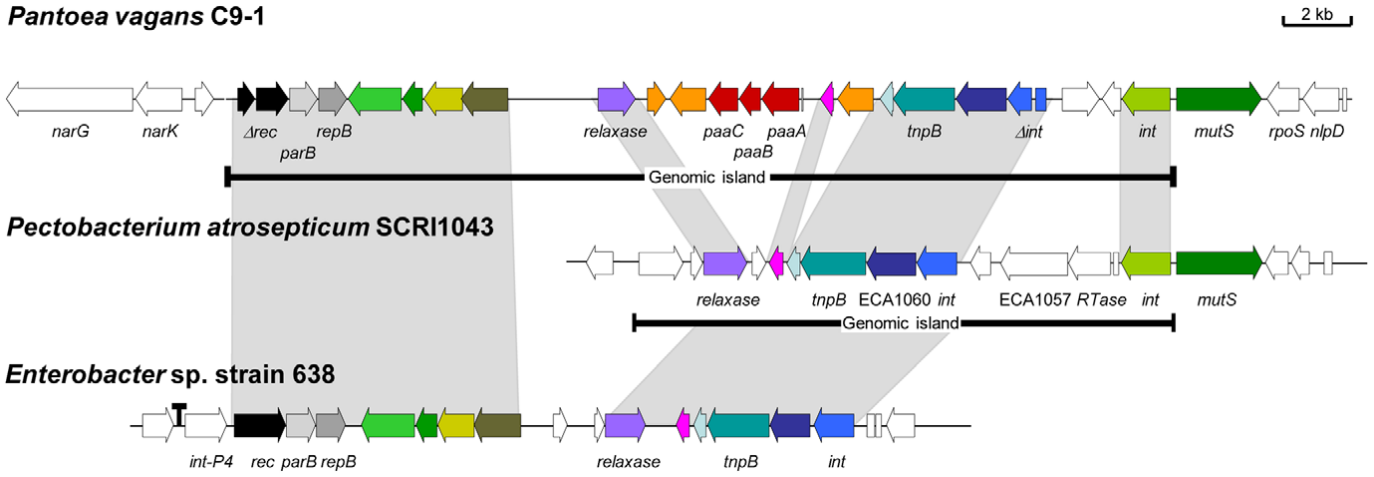
The 28-kb genomic island in the chromosome of *P. vagans* C9-1 containing the pantocin A biosynthetic genes and comparable genomic islands in other organisms. Grey connectors show regions that are conserved between the different organisms, while identical colors of genes indicate genes encoding orthologous proteins. The left and right borders of the genomic islands are indicated.

On the chromosome, a gene cluster (*paaPABC*) encoding the pantocin A biosynthesis and autoresistance genes [24], [25] was identified with 99–100% identity to those of *P. agglomerans* biocontrol strains Eh318 and Eh252 [26]. An antibiotic, previously called herbicolin O [17], with identical chemical characteristics as pantocin A is produced by *P. vagans* C9-1 (C.A. Ishimaru, personal communication). The genes within the *paaPABC* operon have a lower G+C content (40.5%) than the whole genome (55.1%), suggesting a horizontal gene transfer event as origin. This operon resides in a 28 kb genomic island (GI) that was identified by its lower G+C content (37.3%). One border of the GI was integrated in the *N*-terminal region of the *mutS* gene (Fig. 1). On the other border of the GI, a 52 bp repeat was found with high sequence identity to the *N*-terminus of *mutS*. The identified integrase was located adjacent to, but in the opposite direction of *mutS*. Within the GI, other mobile element-related genes were found (i.e., integrases, recombinases, relaxase, *parB* and *repB*), several of them inactivated by frame-shifts. The GI is absent in three other *P. vagans* strains (including the type strain, LMG 24199^T^) but is present in a few other *Pantoea* strains [15].

### Biosynthesis of the antibiotic dapdiamide E

*P. vagans* C9-1 was reported to produce a second antibiotic, called herbicolin I [17], but lately renamed dapdiamide E [27]. The chemical structure was described recently for *P. agglomerans* 48b/90 with the indication that *P. vagans* C9-1 also produced this compound [28], but the biosynthetic genes of this antibiotic were not identified. Dapdiamide E-deficient mutants were obtained using plasposon mutagenesis [29]. Plasmid rescue and sequencing of the insertion site in mutant CIR624 indicated that the insertion took place in a hypothetical gene, encoded on pPag2 in a cluster containing several predicted biosynthetic and hypothetical genes. Analysis of the insertions that are present in the proposed dapdiamide E biosynthetic cluster is underway [27].

### Exopolysaccharide production

Biosynthetic genes for an O-antigen-type exopolysaccharide were found in the genome of *P. vagans* C9-1. The encoded proteins have between 78.5% and 93.4% protein sequence identity with the stewartan gene cluster of *P. stewartii* subsp. *stewartii* and share similar operon organization [30]. The biosynthetic clusters for the exopolysaccharide amylovoran of *E. amylovora* CFBP 1430 and *E. pyrifoliae* DSM 12163^T^ that is involved in pathogenicity, are less related and deviate in the specific glycosyltransferases [6], [7], [31].

### Production of the plant hormone indole acetic acid

**Figure 2:**
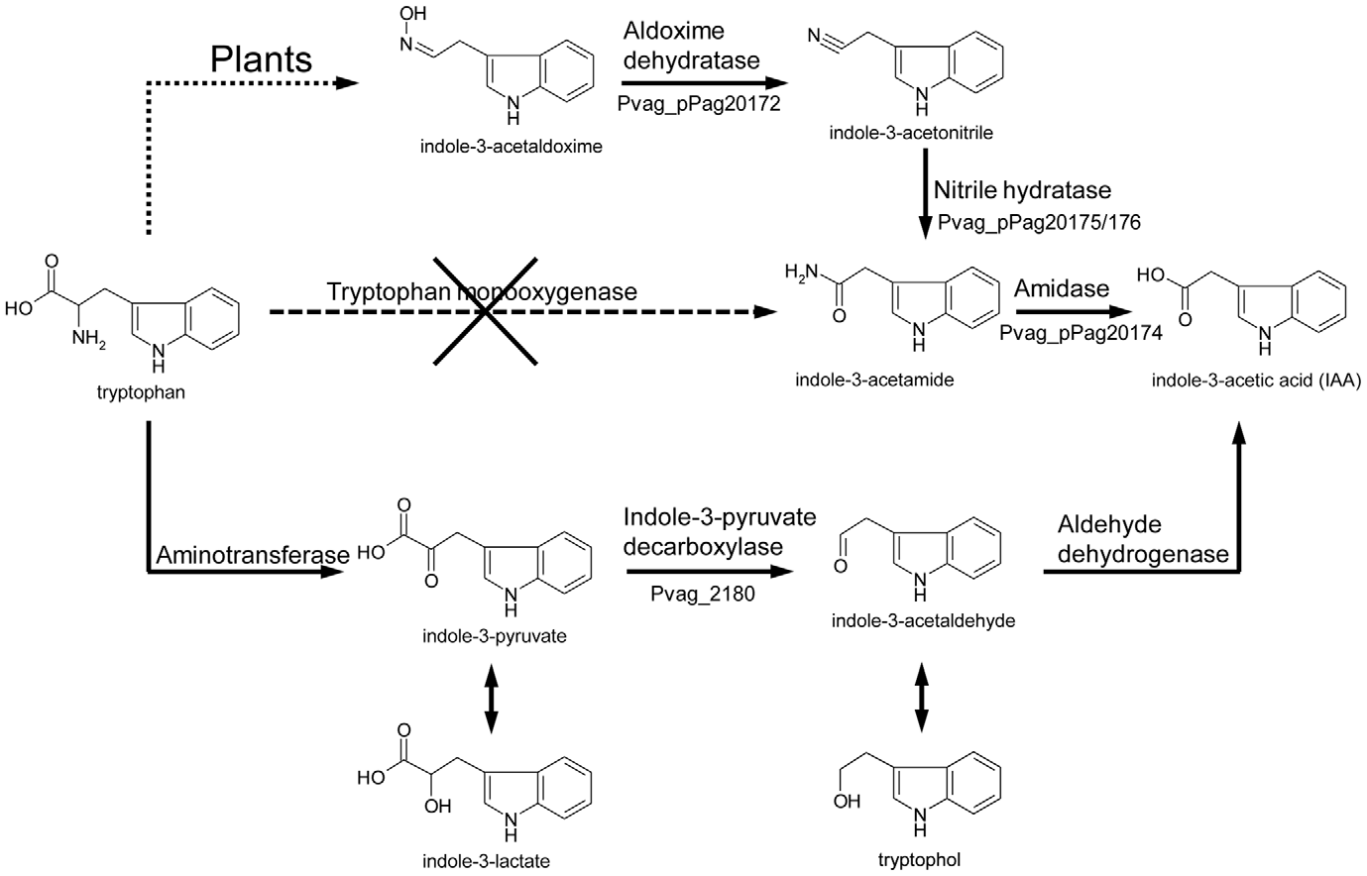
Indole-3-acetic acid (IAA) biosynthetic pathways starting from L-tryptophan. Top: aldoxime pathway; middle: indole-3-acetamide pathway; bottom: indole pyruvate pathway (IpyA). Locus tags are indicated for genes that could be identified. Dashed, crossed-out lines represent pathways that are absent in *P. vagans* C9-1.

The plant growth regulator indole-3-acetic acid (IAA) is produced by some strains of *P. agglomerans* [32], [33]. We confirmed that *P. vagans* C9-1 produces IAA in liquid cultures. Based on the standard curve published by Lindow et al. [33], the concentrations produced by *P. vagans* C9-1 vary roughly between 5 and 10 mg l^−1^, in the same range as IAA produced by the *P. agglomerans* strains tested in their study. On the chromosome of *P. vagans* C9-1, a gene encoding the indole-3-pyruvate (IPyA) decarboxylase IpdC was identified. This protein is the key enzyme of the IPyA pathway in the biosynthesis of IAA [34] (Fig. 2). Another IAA biosynthetic pathway, the indole-3-acetamide pathway that involves tryptophan monooxygenase (Fig. 2), encoded on the plasmid pPATH from phytopathogenic *P. agglomerans* strains [35], [36], is absent in the genome of *P. vagans* C9-1. Additionally, plasmid pPag2 in *P. vagans* C9-1 carries a gene cluster for biosynthesis of IAA from plant-derived aldoximes [34]. The genes for an aldoxime dehydratase, amidase and nitrile hydratase were found in a single gene cluster, similar to the arrangement in *Pseudomonas syringae* pv. *syringae* [37].

### Siderophore production

Siderophore-mediated iron acquisition is essential to overcome conditions of iron limitation encountered on floral tissues [38]. The pathogen *E. amylovora* utilizes desferrioxamine for iron acquisition and this siderophore is implicated in pathogenicity on rosaceous plants [39]. *P. vagans* C9-1 may compete with the pathogen for the iron on flowers by the biosynthesis of high-affinity siderophores.

*P. vagans* C9-1 produces both enterobactin and hydroxamate siderophores [40]. On the chromosome of *P. vagans* C9-1, a complete gene cluster for the production and export of an enterobactin-like siderophore is present. This feature is shared with many members of the *Enterobacteriaceae* [40], but a similar gene cluster is absent in the genomes of the related *P. ananatis* LMG 20103 [41] or all *Erwinia* species [6], [7].

Of the hydroxamate siderophores, desferrioxamine E is the major product of *P. vagans* C9-1, while smaller amounts of desferrioxamines D_2_ and B are produced [40], [42]. On plasmid pPag3, a gene cluster (*dfoJACS*) was found [43] that is related to the *dfoJACS* cluster of *P. ananatis* LMG 20103 [41] with the difference that the latter strain also encodes the TonB-dependent receptor FoxA directly downstream of *dfoS*. Lower sequence identities exist to the *dfoJAC* clusters of *Erwinia* spp. [6], [7] and to lesser extend to the *desABCD* biosynthetic gene cluster for desferrioxamine E in *Streptomyces coelicolor* M145 [44]. In *P. vagans* C9-1, the ferrioxamine receptor gene *foxA* is located on the chromosome.

A striking difference is the number of TonB-dependent siderophore receptors that are present in *P. vagans* C9-1 in comparison to the low number of these receptors in *Erwinia* spp. The genome of *P. vagans* C9-1 encodes 10 TonB-dependent receptors [18], whereas the *Erwinia* spp. only encode four [6], [7]. We postulate that *P. vagans* C9-1 may be a more effective competitor for iron compared to other *Erwinia* spp.

### Type VI secretion systems

**Figure 3:**
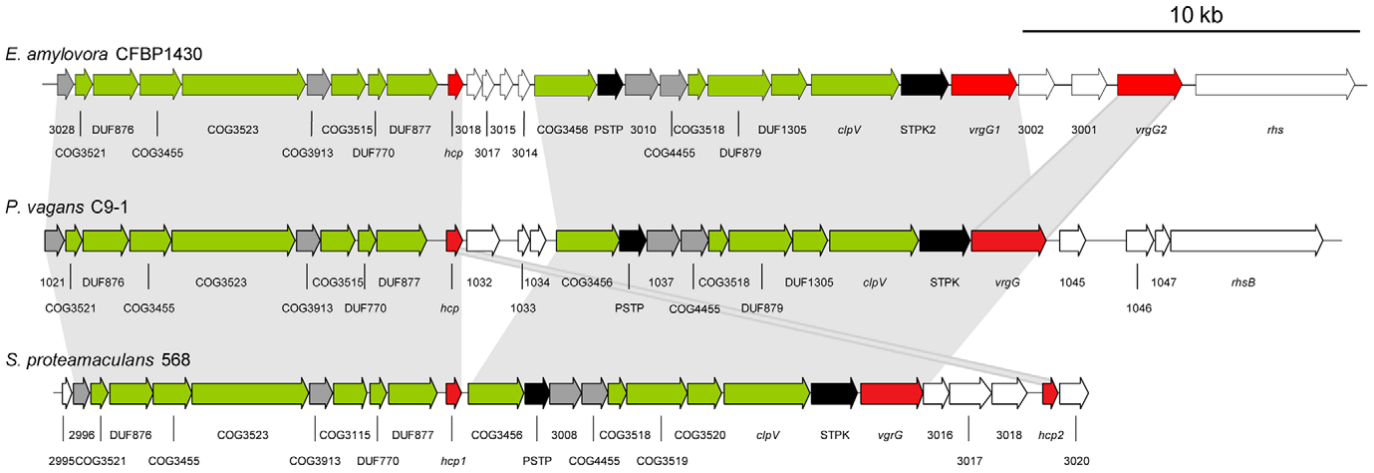
Comparison of type VI secretion system (T6SS) cluster 1 of *P. vagans* C9-1 with comparable regions from *Erwinia amylovora* CFBP 1430 and *Serratia proteamaculans* 568. Blocks of related genes are shaded grey. Putative core genes are colored green, putative effectors red, putative signal transducers black, conserved genes between clusters grey and genes without related or homologues in all other clusters white.

Two type VI secretion system (T6SS) gene clusters were identified in *P. vagans* C9-1. The T6SS cluster 1 has a similar gene organization as those in the closely related enterobacteria *E. amylovora* CFBP 1430 [6] and *S. proteamaculans* 568 (Fig. 3), but differs in the number of putative effector genes (i.e., *hcp* and *vgrG*). A third T3SS cluster as identified in the genomes of the related species *P. ananatis* LMG 20103 [41] and *E. amylovora* CFBP 1430 [6] was not observed.

As several T6SSs have been identified in many pathogenic species (e.g., *Pseudomonas aeruginosa*, *Burkholderia* spp.), it has been hypothesized that they may play a role in host interactions and virulence [45], [46]. T6SSs are present in genome sequences of many non-pathogenic bacteria and studies on the role of T6SSs as a host-targeting virulence factor have yielded inconsistent data [47], [48]. A different role of T6SSs is being considered, that they are involved in inter-bacterial interactions [48]. As such, the T6SSs may impact biocontrol via direct contact between *P. vagans* C9-1 and *E. amylovora*. This role is currently under investigation (Kamber, Smits and Duffy, unpublished).

### Resistance factors

Plasmid pPag2 contains a functional tellurite-resistance operon similar to that of *E. coli* O157:H7 isolates [49]. *P. vagans* C9-1 is resistant to tellurite at 50 µg ml^−1^ and single colony mutants appear at higher concentrations. Plasmid pPag3 encodes a β-lactamase Bla and its cognate regulator AmpR, [43] conferring *P. vagans* C9-1 resistance to ampicillin (200 µg ml^−1^). Additionally, at least 41 genes in the genome of *P. vagans* C9-1 encode putative MFS or DMT super-family multidrug exporters (19 proteins with Transport Classification database (TCDB, [50]) numbers TC 2.A.1.2.-, 9 TC 2.A.1.3.-, and 13 TC 2.A.7.-.-).

The commercialized product BlightBan C9-1S contains a spontaneous rifampicin- and streptomycin-resistant variant of the sequenced wild-type strain. Streptomycin resistance in this strain was identified to be conferred by a single A→G point mutation in the *rpsL* gene, yielding the known mutation K43R that leads to high resistance against the antibiotic. This is analogous to the position and extent of resistance found in streptomycin-resistant strains of *E. amylovora* [9].

### Pathogenicity of *P. vagans* isolates on *Eucalyptus* species

The type strain of *P. vagans* and other initial strains of the species were isolated from *Eucalyptus* leaves showing symptoms of bacterial blight and dieback [14], but the species description did not state whether the strains are the causal agent of the disease. We tested pathogenicity of this species on seedlings of three *Eucalyptus* species. Symptoms of bacterial blight were not observed on *Eucalyptus* inoculated with *P. vagans* strains C9-1, LMG 24199^T^ or LMG 24196; inoculated plants looked similar to water-treated controls. Necrotic lesions were observed on leaves of each *Eucalyptus* species inoculated with high doses of *P. vagans* strain LMG 24195 within 2 weeks after inoculation, but symptoms were not observed when strain LMG 24195 was introduced into petioles or leaves at a lower dose (1×10^6^ CFU ml^−1^). Some strains of *P. vagans*, including C9-1, did not cause any disease symptoms and one strain caused minor symptoms, so pathogenicity towards plants among all strains of *P. vagans* should not be assumed based on isolation of the type strain from diseased tissues.

### Comparative genomics to the related plant pathogen *P. ananatis* LMG 20103

**Figure 4:**
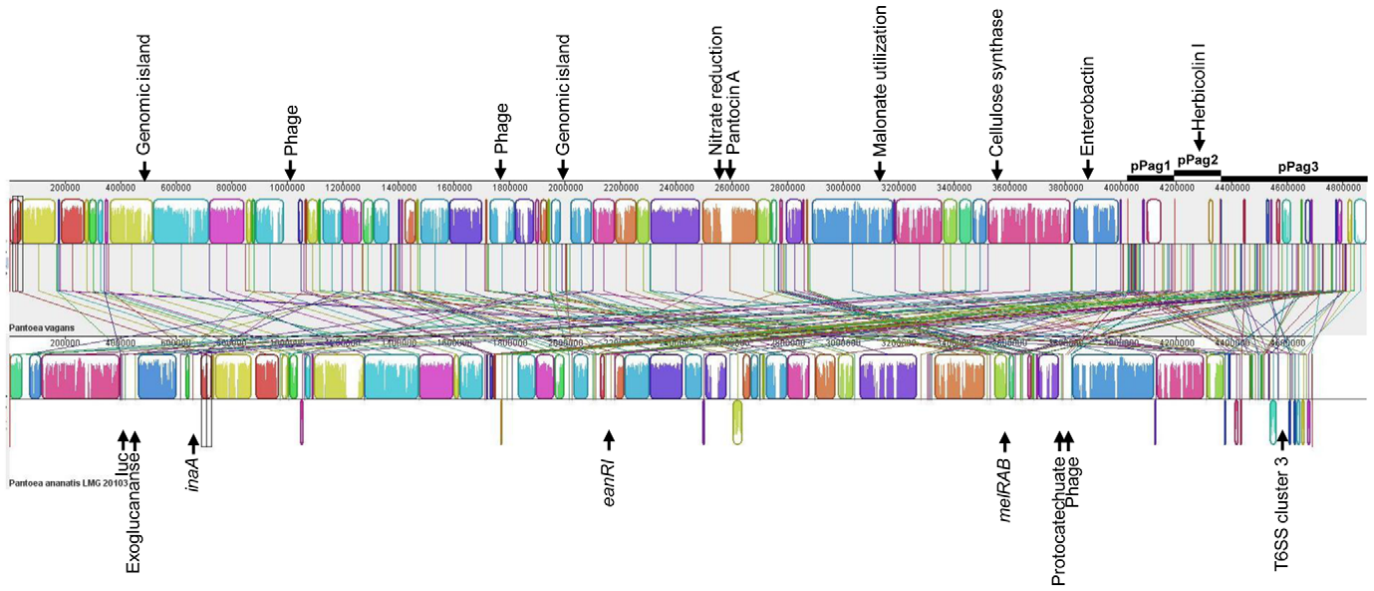
Mauve alignment of the genomes of *P. vagans* C-1 and *P. ananatis* LMG 20103. Selected differential features are indicated.

Recently, the genome of the closely related plant pathogen *P. ananatis* LMG 20103 was published [41], [51]. The most obvious difference between genomes of *P. vagans* C9-1 and *P. ananatis* LMG 20103 is the absence of large plasmids in the latter strain, while the total genome size is comparable. The order on the chromosome is highly syntenic (Fig. 4), although many small, collinear blocks identified on the three plasmids of *P. vagans* C9-1 are scattered over the chromosome of *P. ananatis* LMG 20103. Notably, only one small collinear block was identified on plasmid pPag2, corresponding to the genes encoding sorbitol metabolism in *P. ananatis* LMG 20103 [15], [52].

## Discussion

### Biocontrol activity and genomics reveal main mechanisms

The genome sequence of *P. vagans* strain C9-1 [18] has allowed the identification of several factors that might be involved in biocontrol efficacy. As with most biocontrol agents, preemptive exclusion and nutrient competition with the pathogen for necessary resources are important mechanisms of action [10], [19]. The substrates utilized by *P. vagans* C9-1 include the known nectar sugars (e.g., glucose, sucrose and fructose) [53] and sorbitol, the major transport sugar in *Rosaceae* host plants [54], indicating that *P. vagans* C9-1 likely competes with *E. amylovora* for these substrates at colonization and infection sites [20]. An additional trait of *P. vagans* C9-1 is its production of a variety of antimicrobial metabolites that contribute to biocontrol efficacy, including siderophores and the antibiotics dapdiamide E and pantocin A [17], [24], [25]. Understanding the genetics behind biocontrol antibiotic biosynthesis may enable development of application and/or formulation strategies that optimize expression and preclude inhibition/degradation by co-inoculated or environmental bacteria [55], [56].

### Plasmid pPag2: a plasmid contributing to biocontrol

The biocontrol features dapdiamide E biosynthesis and sorbitol metabolism are located on the *P. vagans* C9-1 plasmid pPag2. This plasmid is not present in the other *P. vagans* strains and was not detected in several *P. agglomerans* strains [43]. The plasmid itself contains several remnants of IS elements and has a mosaic structure. This plasmid could thus constitute a plasmid specific to the biocontrol strain. The other two plasmids of *P. vagans* C9-1 contain general metabolic features of *P. vagans* (e.g., maltose and sucrose utilization) [14] and those genes could be detected in other members of the species by PCR [43]. These plasmids can thus be regarded as indigenous to the species.

### Environmental fitness

We have identified several factors that are involved in environmental fitness, like pigments, siderophores, acyl-homoserine lactones, IAA biosynthesis from aldoximes, tellurite resistance and exopolysaccharides [18], [43]. The pigment zeaxanthine diglucoside is produced by *P. vagans* C9-1 [57], giving it potential protection against reactive oxygen species that are produced during epiphytic growth on sunlight exposed plant surfaces [58]. The siderophores are involved in uptake of iron under conditions of iron limitation occurring on floral surfaces [38], [59], but the presence of numerous TonB receptors also allows competition with other organisms that have similar systems [60]. The biosynthesis of the high-affinity iron siderophore enterobactin and the ability to utilize an array of other iron siderophores not produced by the organism would give *P. vagans* C9-1 a strong competitive advantage over *Erwinia* spp., as the latter group of organisms is able only to synthesize the siderophore desferrioxamine and contains only a limited number of siderophore uptake systems [6], [7]. *P. vagans* C9-1 produces acyl-homoserine lactones [43], [61], a potential quorum-sensing regulation system that might be coupled to environmental fitness factors. The production of exopolysaccharides [30] can protect bacteria against environmental stress like desiccation.

### Potential risk factors evaluated

*P. vagans* C9-1 and other *P. vagans* strains were evaluated for their pathogenicity against Eucalyptus plants. Typical symptoms of bacterial blight were not observed on several *Eucalyptus* species, we conclude that an association of *P. vagans* as the causal agent of bacterial blight of Eucalyptus [14] is not confirmed. In addition, *P. vagans* C9-1 is not pathogenic to pome fruit flowers and does not cause a hypersensitive response when infiltrated into tobacco leaves (V.O. Stockwell, personal communication). Additional virulence factors like the type III secretion systems (T3SS) and effectors of *P. agglomerans* pathovars *gypsophilae* or *betae* [36] were not identified on the chromosome. Essentially this confirms that *P. vagans* C9-1 is not a plant pathogen.

The close phylogenetic position of *P. vagans* to *P. agglomerans* generates problems when identifying this species using some commercial systems [14], [15], [16], [62]. The irregular, and often inaccurate, identification of isolates from clinical samples as “*P. agglomerans*” [15], [16] has resulted in both species being classified as BL-2 pathogens, even though clinical assumptions are never supported by attempts to fulfill Koch's postulates, and no evidence has ever been presented demonstrating toxicity, pathogenicity or allergenicity for either species [15]. We confirm that no known or putative genes involved in pathogenicity to animals, humans, plants or other organisms (e.g., T3SS, toxins) are found in the genome and there is no evidence that *P. vagans* C9-1 interacts with or persists in mammals (http://www.epa.gov/opp00001/biopesticides/ingredients/tech_docs/brad_006470.pdf). Several environmental organisms like *E. amylovora* or *S. proteamaculans* have orthologous gene clusters for the T6SSs of *P. vagans* C9-1 and its role is proposed to be involved in inter-bacterial interactions [48]. The presence of a non-transferable point-mutation conferring streptomycin resistance in the commercialized strain C9-1S allows the combinatorial treatment with the antibiotic without suppressing the growth of populations of *P. vagans* C9-1 on the treated plants [63].

## Materials and Methods

### Strains, media and growth conditions

*Pantoea vagans* C9-1 was isolated from stem tissue of a *Malus*×*domestica* ‘Jonathan’ in Michigan, USA [17] and evaluated over the past 20 years for biological control of fire blight [8]. A spontaneous streptomycin- and rifampicin-resistant mutant was approved and registered by the USA EPA in 2007 under the trade name BlightBan C9-1S (NuFarm Americas, Burr Ridge, IL, USA) in the USA and Canada. Additional *P. vagans* strains (LMG 24195, LMG 24196 and LMG 24199^T^) were obtained from T.A. Coutinho (FABI, University of Pretoria, South Africa). Bacteria were grown on LB medium [64] at 28°C. Catabolism assays were done in M9 minimal medium [64] supplemented with carbon sources (e.g., glucose, sorbitol, maltose or sucrose).

### Metabolic profiling

The metabolic profile of *P. vagans* C9-1 was assayed using Biolog GN2 and AN plates. Pre-cultures were grown in M9 medium [64] with 5 mM glucose and allowed to grow to late stationary phase to ensure complete substrate utilization. The cells were washed once and re-suspended in fresh M9 medium. Attenuance at 600 nm (A_600_) was set to 0.15 per well, 100 µl of inoculum was added. The plates were visually interpreted after incubation for 1, 2 and 5 days at 28°C. Additional data on the metabolism of nutrient sources for *P. vagans* C9-1 were generated in replicate plates incubated at 22°C by the Biolog Phenotype Microarray Services (Hayward, CA, USA). Further information and comments on nutrient sources is provided in supporting information (Text S1).

### Indole acetic acid biosynthesis assays

For production of indole acetic acid (IAA) by *P. vagans* strains, the method of Lindow et al. [33] was followed. Briefly, three replicate cultures per strain were grown in KB broth amended with 0.2 mg ml^−1^ L-tryptophan for 48 h at 27°C. Cultures were harvested by centrifugation (5 min, 14,000 rpm) and 1 ml culture supernatant was added to 2 ml of reagent (2% 0.5 M FeCl_3_ in 35% perchloric acid) [65]. The samples were incubated at room temperature for 30 min, and OD_530 nm_ was measured. As reference, non-inoculated broth plus reagent was used. As controls for IAA production, cultures of a known IAA producer (*P. agglomerans* strain 299R) and known non-producers (*Pseudomonas fluorescens* A506 and *Erwinia amylovora* Ea153) were included. This experiment was conducted twice with similar results.

### Pathogenicity tests on *Eucalyptus*

*P. vagans* strains C9-1, LMG 24199^T^, LMG 24195 and LMG 24196 were cultured for 2 days on LB agar at 27°C. Cells were removed from the surface of the agar and suspended in sterile distilled water; cell concentration was adjusted with a spectrophotometer. *Eucalyptus grandis*, *E. gunnii* and *E. nitens* plants were propagated from seeds kindly provided by Windmill Outback Nursery, VA, USA and maintained in a greenhouse. Leaves and petioles of five replicate 3-month old plants were inoculated with bacterial suspensions (1×10^6^ and 1×10^8^ CFU ml^−1^) by the methods of Coutinho et al. [66] and covered with plastic bags to maintain humid conditions. Additional plants were inoculated by cutting leaves transversely with scissors dipped in 1×10^9^ CFU ml^−1^. Control plants for each method were treated with sterile water. Plants were examined for symptoms of bacterial blight (i.e., water-soaking, necrosis, wilt and/or leaf abscission) periodically over 3 weeks. The experiment was repeated three times with similar results.

### Genome annotation, comparative genomics and metabolic reconstruction

Genes were predicted using a combined strategy [67] based on the CDS prediction programs Glimmer [68] and Critica [69]. Subsequently, the potential function of each predicted gene was automatically assigned using the GenDB annotation pipeline [70]. The resulting genome annotation was manually curated, and metabolic pathways were identified using the KEGG pathways tool [71] in GenDB. Transport proteins were classified according to the nomenclature in the Transporter Classification Database [50].

Routine sequence manipulations were done using the programs of the Lasergene package (DNASTAR, Madison, WI, USA). Whole-genome comparisons were done using the progressive alignment option of the Mauve comparison software (Version 2.0 [72]).

The genome sequence of *P. vagans* C9-1 was compared to those of *P. ananatis* LMG 20103 and *E. amylovora* CFBP 1430 to identify the set of common genes composing the core genome for this genus and the set of genes unique to each species, referred to as singletons. For this purpose, an “all-against-all” comparison of the genes was accomplished using the BLAST alignment tool [73]. The genes were aligned based on the protein sequence (BLASTP) with an initial *e*-value cut-off of 1E-5 using the BLOSUM62 scoring matrix. Genes were considered orthologous when a reciprocal best BLAST hit was found between two genes, and when both BLAST hits were based on alignments exceeding 70% sequence identity spanning over at least 70% of the query gene length [74]. The *Pantoea* genus core genome was calculated as the set of genes of a reference strain for which an orthologous gene could be found in each of the compared genomes. In contrast, genes of one strain were considered to be singleton genes when they had no BLAST-hits comparability with any of the other genomes that satisfied the given criteria [74].

## Supporting Information

Table S1 Carbon sources for *P. vagans* C9-1, as determined with Biolog plates GN2, AN or the Biolog PM system plates PM1 and PM2A.
(DOC)

Text S1 Further information and comments on nutritional sources and substrates.
(DOC)
