# Supplementary material for: Metabolic Versatility and Antibacterial Metabolite Biosynthesis Are Distinguishing Genomic Features of the Fire Blight Antagonist Pantoea vagans C9-1
Source: PLoS One. 2011 Jul 15;6(7):e22247. doi: 10.1371/journal.pone.0022247 (PMC3137637; doi:10.1371/journal.pone.0022247)
Supplement: Table S1 — Carbon sources for P. vagans C9-1, as determined with Biolog plates GN2, AN or the Biolog PM system plates PM1 and PM2A. (DOC) [file pone.0022247.s001.doc]

|  | **Biolog**  **GN2^a^** | **Biolog**  **AN^a^** | **Biolog PM1 and PM2A^b^** | **Brady et al., 2009^c^** | **Pathway identified** |
| --- | --- | --- | --- | --- | --- |
| **Sugars and -derivates** |  |  |  |  |  |
| *N*-acetyl-D-glucosamine | 1 | 1 | 3.3 |  | *nagABCDE* |
| L-arabinose | 1 | - | 3.5 | Yes | *araABFGHC* |
| arbutin | - | 1 | 1.9 |  | *ascBGH, bglBGH* |
| D-cellobiose | 5 | 5 | 1.4 | Yes | *ascBGH, bglBGH* |
| 2-deoxy-D-ribose | - | - | 1.4 |  | *rbsK* |
| D-fructose | 1 | 1 | 2.2 | Yes | *fruABK* |
| D-fructose-6-phosphate | - | - | 2.3 |  |  |
| D-galactose | 1 | 1 | 3.3 | Yes | *mglSBAC, galP, galR, galTKM* |
| L-galactonic acid-γ-lactone | - | - | 4.2 |  |  |
| 3-O-β-D-galactopyranosyl-D-arabinose | - | - | 1.5 |  |  |
| D-galacturonic acid | 1 | 1 | 5.1 |  | *uxaCBA* |
| α-D-glucose | 1 | 1 | 2.3 | Yes | *ptsG, crr, glycolysis* |
| glucose-1-phosphate | 1 | 1 | 3.0 |  | *glycolysis* |
| glucose-6-phosphate | 1 | 1 | 2.5 |  | *glycolysis* |
| D-glucosamine | - | - | 2.9 |  |  |
| D-gluconic acid | 1 | 1 | 3.8 |  | *gntUKR* |
| D-glucuronic acid | 1 | 1 | 3.5 |  | *uxaCBA, exoTR* |
| glucuronamide | 1 | - | 1.4 |  |  |
| m-inositol | 1 | 1 | 3.8 | Yes | *iolEGD, iolC, iolB, iolI* |
| 5-keto-D-gluconic acid | - | - | 1.3 | Variable | *kdgK, kguT* |
| L-lyxose | - | - | 2.6 |  |  |
| maltose | 1 | 1 | 2.5 | Yes | *malGFEKLMQPT* |
| maltotriose | 1 | 1 | 3.0 | Yes | *malGFEKLMQPT* |
| D-mannitol | 1 | 1 | 2.8 | Yes | *mltADR* |
| D-mannose | 1 | 1 | 2.9 | Yes | *manAXYZ* |
| β-methyl-D-galactoside | - | 2 | 0.8 |  |  |
| β-methyl-D-glucoside | 1 | 1 | 2.2 |  |  |
| mucic acid (D-galactarate) | - | - | 3.9 |  | *yhaG* |
| L-rhamnose | 1 | 1 | 3.5 | Yes | *rhaDABSRT* |
| D-ribose | - | - | 2.5 | Yes | *rbsDACBKR* |
| D-saccharic acid (D-glucarate) | 1 | 1 | 4.5 |  | *ygcZYXyhaFED* |
| salicin | - | 1 | 1.8 |  | *ascBGH, bglBGH* |
| D-sorbitol | 1 | 1 | 2.8 | No | *srl(AEBDMRQ)_1_, slr(AEBDMRQ)_2_* |
| sucrose | 1 | 1 | 2.3 | Yes | *scrRABY* |
| D-trehalose | 1 | 1 | 2.8 | Yes | *treA, otsBA, treF* |
| D-xylose | - | - | 2.8 | Yes | *xylBAFGHRE* |
|  |  |  |  |  |  |
| **Organic acids and derivates** |  |  |  |  |  |
| acetoacetic acid | - | - | 1.5 |  |  |
| γ-aminobutyric acid | 1 | - | 3.6 |  | *gabPT* |
| bromosuccinic acid | 1 | - | 2.6 |  |  |
| cis-aconitic acid | 1 | - | - | Yes |  |
| fumaric acid | - | 1 | 4.5 | Yes |  |
| D,L-lactic acid | 1 | 1 | 2.3 |  | *dld, lldPRD* |
| L-lactic acid | - | 1 | - |  | *lldPRD* |
| D-lactic acid methyl ester | - | 5 | 1.9 |  |  |
| D,L-malic acid | - | - | 4.8 |  |  |
| D-malic acid | - | 5 | 4.0 |  |  |
| L-malic acid | - | 1 | 5.2 |  |  |
| malonic acid | 5 | - | 0.6 | Yes |  |
| methyl pyruvate | 1 | 1 | 1.6 |  |  |
| pyruvic acid | - | 1 | 2.9 |  |  |
| succinic acid | 1 | 2 | 3.4 | Yes |  |
| L-tartaric acid | - | - | 2.9 | Yes | *ttuABC* |
| urocanic acid | 2 | 5 | - |  | *hutUHCFIG* |
|  |  |  |  |  |  |
| **Amino acids** |  |  |  |  |  |
| D-alanine | 5 | - | 2.0 | Yes |  |
| L-alanine | 2 | 5 | 4.1 | Yes |  |
| L-alanyl-L-glutamine | - | 5 | - |  |  |
| L-alanyl-L-histidine | - | 5 | - |  |  |
| L-alanylglycine | 5 | - | 1.9 |  |  |
| L-asparagine | 5 | 5 | 4.5 |  |  |
| L-aspartic acid | 5 | - | 1.9 | Yes |  |
| L-glutamic acid | 5 | 5 | 4.3 | Yes |  |
| L-glutamine | - | 5 | 4.5 |  |  |
| glycyl-L-aspartic acid | 5 | 5 | 1.9 |  |  |
| glycyl-L-glutamic acid | 5 | - | 2.2 |  |  |
| glycyl-L-glutamine | - | 5 | - |  |  |
| glycyl-L-proline | - | 5 | 2.5 |  |  |
| L-histidine | 2 | - | 2.4 | No | *hutUHCFIG* |
| L-ornithine | 5 | - | 1.2 | Yes |  |
| L-proline | 2 | - | 3.4 | Yes |  |
| L-pyroglutamic acid | 5 | - | 0.5 | No |  |
| L-serine | 2 | 5 | 3.1 | Yes |  |
| L-valine plus L-aspartic acid | - | 5 | - |  |  |
|  |  |  |  |  |  |
| **Polymers** |  |  |  |  |  |
| dextrin | 1 | 5 | 1.9 |  | *malGFEKLMQPT* |
| pectin | - | - | 2.5 |  | Pectinases, *ganKEFGABCLR* |
|  |  |  |  |  |  |
| **Other compounds** |  |  |  |  |  |
| adenosine | - | - | 3.3 |  |  |
| inosine | 1 | 5 | 2.2 |  |  |
| glycerol | 1 | 1 | 2.4 | Yes | *glpFKX* |
| D,L-α-glycerolphosphate | 1 | 1 | 2.1 |  | *glpT* |
| dihydroxy acetone | - | - | 1.9 |  |  |

^a^ A number indicates the number of days after inoculation that a well showed a strong reaction. Weaker reactions can already be observed earlier with some of the substrates.

^b^ Fold change compared to the average negative control. Data are combined from two individual reads.

^c^ Substrates mentioned by {Brady, 2009 #418} as substrates utilized by *P. vagans* strains.
