# Supplementary material for: Metabolic Versatility and Antibacterial Metabolite Biosynthesis Are Distinguishing Genomic Features of the Fire Blight Antagonist Pantoea vagans C9-1
Source: PLoS One. 2011 Jul 15;6(7):e22247. doi: 10.1371/journal.pone.0022247 (PMC3137637; doi:10.1371/journal.pone.0022247)
Supplement: Text S1 — Further information and comments on nutritional sources and substrates. (DOC) [file pone.0022247.s002.doc]

*Carbon sources additional comments*

The data on carbon source utilization are given in the Supplemental Information Table S1, including the typical utilization of carbon sources by *P. vagans* [Brady et al., 2009]. There are some differences compared to the literature. *P. vagans* C9-1 is, in contrast to other *P. vagans* strains [Brady et al., 2009], able to utilize sorbitol, a plasmid-encoded trait in C9-1, L-histidine and L-pyroglutamic acid. This strain can utilize 5-keto-D-gluconic acid (variable for the species *P. vagans* [Brady et al., 2009]). For the utilization of 5-keto-D-gluconic acid and L-histidine, the genes were identified in the genome, whereas it is expected that a hydrolase is needed for the conversion of L-pyroglutamic acid. The comparison to *P. agglomerans* strains shows good agreement between the data and literature [Gavini et al., 1989], with the exception of sorbitol, that is also a rare trait in *P. agglomerans* [Rezzonico et al., 2009].

***Nitrogen sources additional comments***

Additional to ammonia, nitrate, nitrite, urea, uric acid and biuret are utilized as nitrogen sources. Nitrate and nitrite can be taken up and transformed using the gene products of the *narIJHGKL* gene cluster. Furthermore, all nucleobases and many amino acids (L-Ala, L-Arg, L-Asn, L-Asp, L-Cys, L-Glu, L-Gln, Gly, L-His, L-Phe, L-Pro, L-Ser, L-Trp, L-Tyr, D-Ala, D-Asp, L-Citrulline, L-Ornithine) were identified as alternative nitrogen sources. Several of the amino acids utilized as nitrogen source cannot serve as a carbon source for *P. vagans* C9-1 (Supplemental Information, Table S1). This phenomenon has rarely been studied.

Many di- and tripeptides can serve as nitrogen source for *P. vagans* C9-1. This indicates that the many transporters of the PepT family (TC 3.A.1.5.-) may play a role in uptake of these di- and tripeptides. The composition of dipeptides that are not or only poorly utilized largely reflects the utilization of the single amino acids and is independent of the position in the dipeptide. Inclusion of β-alanine or D-amino acids in dipeptides completely prohibits growth.

*Phosphorus sources*

Many of the organic phosphate-esters included on the PM plates can be utilized as phosphorus source for *P. vagans* C-1. Exceptions are phosphonates, even in spite of the presence of the complete *phn* gene cluster on the chromosome. All nucleobase monophosphates are utilized, except for the nucleobase-3’,5’-cyclic monophosphates, which only give weaker growth.

*Sulfur sources*

Sulfate, thiosulfate and tetrathionate were efficiently utilized by *P. vagans* C9-1; thiophosphate and dithiophosphate were supporting only weak growth. The latter two compounds also were weak phosphorus sources, indicating that *P. vagans* C9-1 might be sensitive to these two compounds. With the exception of cysteamine, all cysteine and methionine derivates were utilized. From the tested sulfonates, taurine, hypotaurine, isethionate, methane sulfonate, butylsulfonate, L-cysteate and L-cysteine sulfinate were utilized, most probably using the TauABCD system [Eichhorn et al., 1997; van der Ploeg et al., 2001]. A representative of the aromatic sulfonates, *p*-aminobenzenesulfonate is not a sulfur source, corresponding to the absence of corresponding gene systems [Vermeij et al., 1999] in the genome of *P. vagans* C9-1.

**References**

Brady CL, Venter SN, Cleenwerck I, Engelbeen K, Vancanneyt M, et al. (2009) *Pantoea vagans* sp. nov., *Pantoea eucalypti* sp. nov., *Pantoea deleyi* sp. nov. and *Pantoea anthophila* sp. nov. Int J Syst Evol Microbiol 59: 2339-2345.

Eichhorn E, van der Ploeg JR, Kertesz MA, Leisinger T (1997) Characterization of α-ketoglutarate-dependent taurine dioxygenase from Escherichia coli. J Biol Chem 272: 23031-23036.

Gavini F, Mergaert J, Beji A, Mielcarek C, Izard D, et al. (1989) Transfer of *Enterobacter agglomerans* (Beijerinck 1888) Ewing and Fife 1972 to *Pantoea* gen. nov. as *Pantoea agglomerans* comb. nov. and description of *Pantoea dispersa* sp. nov. Int J Syst Bacteriol 39: 337-345.

Rezzonico F, Smits THM, Montesinos E, Frey JE, Duffy B (2009) Genotypic comparison of *Pantoea agglomerans* plant and clinical strains. BMC Microbiol 9: 204.

van der Ploeg JR, Iwanicka-Nowicka R, Bykowski T, Hryniewicz MM, Leisinger T (1999) The Escherichia coli ssuEADCB gene cluster is required for the utilization of sulfur from aliphatic sulfonates and is regulated by the transcriptional activator Cbl. J Biol Chem 274: 29358-29365.

Vermeij P, Kertesz MA (1999) **Pathways of assimilative sulfur metabolism in *Pseudomonas putida*.** J Bacteriol 181: 5833-5837.
